# Contribution of APC and MUTYH mutations to familial adenomatous polyposis susceptibility in Hungary

**DOI:** 10.1007/s10689-015-9845-5

**Published:** 2015-10-07

**Authors:** Janos Papp, Marietta Eva Kovacs, Zoltan Matrai, Enikő Orosz, Miklós Kásler, Anne-Lise Børresen-Dale, Edith Olah

**Affiliations:** Department of Molecular Genetics, National Institute of Oncology, Rath Gy. u. 7-9, Budapest, 1122 Hungary; Hamon Center for Therapeutic Oncology, University of Texas Southwestern Medical Center, Dallas, TX USA; Department of General Surgery, National Institute of Oncology, Budapest, Hungary; Department of Clinical Central Laboratory, National Institute of Oncology, Budapest, Hungary; Multidisciplinary Centre of Head and Neck Oncology, National Institute of Oncology, Budapest, Hungary; Department of Genetics, Institute for Cancer Research, Oslo University Hospital Radiumhospitalet, Oslo, Norway; Institute of Clinical Medicine, Faculty of Medicine, University of Oslo, Oslo, Norway

**Keywords:** Familial adenomatous polyposis, Colorectal cancer, Germline mutations, APC, MUTYH, Genotype–phenotype correlations

## Abstract

Familial adenomatous polyposis (FAP) is a colorectal cancer predisposition syndrome with considerable genetic and phenotypic heterogeneity, defined by the development of multiple adenomas throughout the colorectum. FAP is caused either by monoallelic mutations in the adenomatous polyposis coli gene APC, or by biallelic germline mutations of MUTYH, this latter usually presenting with milder phenotype. The aim of the present study was to characterize the genotype and phenotype of Hungarian FAP patients. Mutation screening of 87 unrelated probands from FAP families (21 of them presented as the attenuated variant of the disease, showing <100 polyps) was performed using DNA sequencing and multiplex ligation-dependent probe amplification. Twenty-four different pathogenic mutations in APC were identified in 65 patients (75 %), including nine cases (37.5 %) with large genomic alterations. Twelve of the point mutations were novel. In addition, APC-negative samples were also tested for MUTYH mutations and we were able to identify biallelic pathogenic mutations in 23 % of these cases (5/22). Correlations between the localization of APC mutations and the clinical manifestations of the disease were observed, cases with a mutation in the codon 1200–1400 region showing earlier age of disease onset (*p* < 0.003). There were only a few, but definitive dissimilarities between APC- and MUTYH-associated FAP in our cohort: the age at onset of polyposis was significantly delayed for biallelic MUTYH mutation carriers as compared to patients with an APC mutation. Our data represent the first comprehensive study delineating the mutation spectra of both APC and MUTYH in Hungarian FAP families, and underscore the overlap between the clinical characteristics of APC- and MUTYH-associated phenotypes, necessitating a more appropriate clinical characterization of FAP families.

## Introduction

Familial adenomatous polyposis is a rare colorectal cancer (CRC) predisposition syndrome, giving rise to approximately 1 % of all CRC cases and showing a distinctive phenotypic heterogeneity. In its autosomal dominant form (FAP1, OMIM#175100) it is characterized by the presence of hundreds or thousands of adenomas in the large bowel, and also by associated extracolonic manifestations including desmoid tumors, dental and skin abnormalities, retinal spots and malignant tumors of other organs. One of the colorectal polyps usually transforms into carcinoma at an early age [1–4]. A phenotypic variant of FAP1, called attenuated FAP **(**AFAP**)**, shows less aggressive features, fewer (<100) adenomas, 10–15 years later age at disease onset [5–7] and require distinct surveillance and clinical management approaches [8–10].

FAP1 is caused by germline mutations of the adenomatous polyposis coli (APC) gene, encoding a multifunctional gatekeeper tumor suppressor protein expressed in a wide variety of tissues. Constitutional pathogenic mutations are identifiable in 60–80 % of the classic FAP1 cases, but only in 10–30 % of AFAP patients [11–14].

In the past decade, a significant part of patients with (A)FAP without a detectable germline APC mutation have been found to carry biallelic mutations in the base excision repair gene MUTYH, a highly conserved DNA glycosylase involved in the repair of oxidative guanine damage. This discovery led to the description of FAP2 (OMIM#608456, usually known as MUTYH-associated polyposis, MAP), a recessively inherited phenotypic variant of familial adenomatous polyposis, with clinical features overlapping those of AFAP and classical FAP [14–19].

In the present study we examined the first set of (A)FAP patients from Hungary and one of the largest cohort from Central-Eastern Europe [20–25] in order to determine the mutational spectra of the APC and MUTYH genes diagnosed with colorectal polyposis to compare the clinical features of the mutation carriers and also to evaluate the respective roles of these genes in the inherited CRC burden in this Central-Eastern-European population.

## Methods

### Patients and samples

Individuals in this study were referred for genetic counselling and testing to the Department of Molecular Genetics at the National Institute of Oncology (Budapest, Hungary), through a 15-year service (1999–2014). All investigations have been carried out in agreement with internationally recognized guidelines, using study protocols approved by the Institutional Ethical Board. Written informed consent was provided by each patient. Included in this study were 87 patients from well-characterized familial adenomatous polyposis kindreds. The majority of cases presented the clinical symptoms of classical FAP, while 21 patients were diagnosed with <100 polyps and were classified as AFAP. Polyp counts are based on the endoscopic findings of gastroenterologists or on the reports of clinical pathologists. Since collecting family history information is still often neglected in the clinical practice, and self-reporting was also shown to be highly inaccurate [26, 27], reliable family history information gathered through past clinical records was available only for a minority of the patients/families involved. Therefore, this data was not used for the ab initio classification of the families.

### Mutation analysis

DNA was extracted from blood samples of all consenting subjects using either the classic phenol–chloroform method or the ArchivePure DNA Blood Kit (5 Prime). The entire coding region and splice junctions of the *APC* gene were amplified by PCR (primer sequences are available upon request). Mutation screening was performed using direct bidirectional sequencing on an ABI 3130 Genetic Analyzer (Life Technologies). The presence of all mutations was confirmed using a different blood sample. Additionally, the coding region of *APC* was screened for genomic copy number aberrations using the MLPA (multiplex ligation-dependent probe amplification) Kit P043 (MRC-Holland), according to the manufacturer’s recommendations, and as described previously [28, 29].

All patients found negative for deleterious APC gene mutations were screened for the presence of variants in the MUTYH gene, again by PCR amplification and direct bidirectional sequencing of all coding exons and neighbouring splice sites. The biallelic nature of MUTYH variants (i.e. their trans status) was ascertained for cases carrying two different mutations by inspecting their presence/absence in first-degree relatives, whenever such samples were available. Copy number analysis of the gene was performed using the MLPA Kit P378 (MRC-Holland).

The novel or recurrent status of the point mutations was assessed by comparing our data with those available in variant databases: HGMD Professional 2013.3 (release date 27th Sept 2013, http://www.hgmd.cf.ac.uk/); the InSiGHT Colon Cancer Gene Variant Databases [30] (http://chromium.liacs.nl/LOVD2/colon_cancer/home.php, Accessed on May-2014) and the APC mutation database [31] at http://www.umd.be/APC/ (accessed on May-2014).

### Characterization of large deletions

**Fig. 1:**
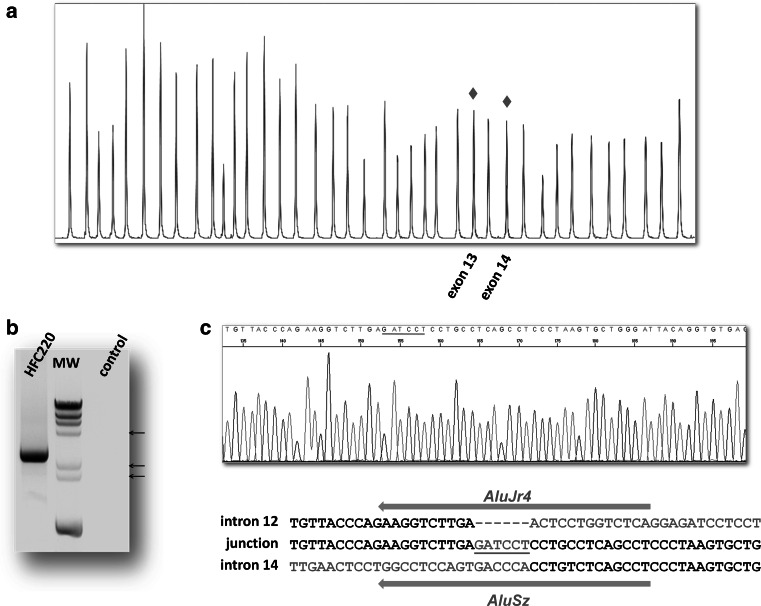
Identification and characterization of the large deletion (c.1627-185_1958+651del7146insGATCCT) in case HFC220. **a** MLPA analysis results of a heterozygous deletion removing exon 13 and 14 of the APC gene. The electropherogram of the patient (*red*) is superimposed on that of a control sample (*blue*). The *peaks* showing 50 % reduction of intensity are marked by *red diamonds*. The agarose gel image of the amplification product with primers located in intron 12 and intron 14 is shown on **b**. PCR was performed using the Multiplex PCR Kit (Qiagen) with short extension time (3.5 min), so the 9866 bp long normal fragment cannot be amplified (empty control lane). Case HFC220 shows a PCR product of approximately 3000 bp. The DRIgest III (GE Healthcare) molecular weight marker (MW) was used for this experiment, the 4.36, 2.32 and 2.03 kb fragments are indicated by *arrows* on the *left side* of the gel image. **c** The sequencing results of the above PCR product with a nested primer, showing the nucleotide sequence around the breakpoints. Red nucleotides are non-templated insertions, *light grey letters* are applied to mark deleted nucleotides. *Grey arrows* on *top* and *below* the sequences show the orientation of the Alu elements involved in the deletion. (Color figure online)

To determine the exact lengths of deletions having both breakpoints within the APC gene, a combination of XL-PCR and sequencing by primer walking was applied, where all deletions required individual approaches in selecting the appropriate PCR cycle settings and primer designs. An example is outlined in the legend of Fig. 1.

Although the precise localization of the deletion breakpoints that extended over the 5′ and/or 3′ gene boundaries has not been determined, gene dosage assays were performed to estimate the lengths of these sequence changes. Twenty-nine regions were used for copy number analyses from 7.6 MB upstream to 1.5 MB downstream of the *APC* gene, all selected from non-repetitive regions. Primer sequences and exact localization data are available upon request from the authors.

**Fig. 2:**
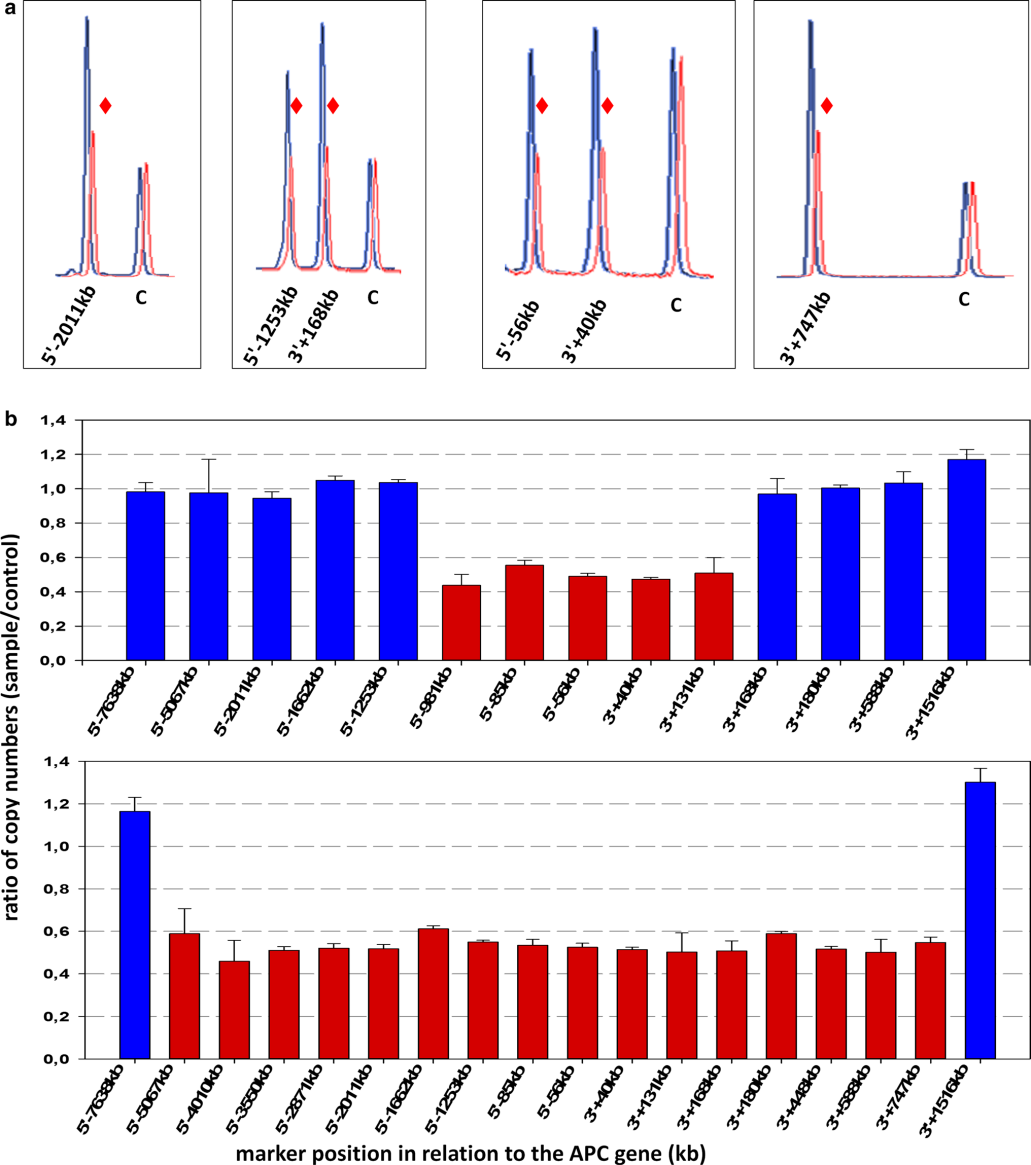
Approximate localization of large genomic deletions by robust dosage PCR (RD-PCR). **a** Examples of copy number determination at several regions in duplex and triplex RD-PCR assays. The electropherograms of the patient (case HFC208, *red*) were superimposed on those of a control sample (*blue*), and shifted slightly to the right for better visibility. The heights of the cntrol peaks (*C*) were adjusted to the same level. The deletion for a given amplicon (names given under each peak, reflecting the position of the given marker) is seen as a ~50 % reduction of peak intensity (*red diamonds*). **b** Diagram showing the approximate size of two deletions (case HFC106: *upper*; and HFC208: *lower*). The distance of the markers from the APC gene is given on the X axis (in kilobases, using *negative numbers* for upstream and *positive numbers* for downstream markers), while *Y axis* indicates the copy numbers normalized for the average of three control samples. A 40–60 % reduction for a given marker indicates the presence of the heterozygous deletion (*red bars*). Not all samples were tested for all positions. (Color figure online)

The first TaqMan-based gene dosage assays in our studies were performed as previously described [32]. At a later stage of our experiments, a different approach was preferred, and copy number determination was done using the robust dosage PCR (RD-PCR) methodology, in which the target locus and an internal control with known copy number are co-amplified [33, 34]. Briefly, PCR was performed in a duplex or triplex design containing primers for the target locus/loci and for an endogenous control with two copies. A touch-down setting was used in the amplification step: after initial denaturation and enzyme activation (95 °C for 15 min), 14 cycles were carried out with a denaturation step for 15 s at 95 °C, 20 s at the annealing temperature (starting from 62 °C and ending at 55 °C with 0.5 °C decrease per cycle), and extension for 30 s at 72 °C. This was followed by another 10 PCR cycles using 55 °C as annealing temperature (Multiplex PCR Kit, Qiagen). In order to decrease inter-individual variation, the DNA samples were heated (90 °C for 10 min) in 2 × TE (pH = 8.0) before adding the PCR mixture [35, 36]. The reaction products were run an Agilent 2100 Bioanalyzer (High Sensitivity DNA Kit, Agilent), and copy number status of the target amplicon was assessed by comparing ratio-of-yield measures for input templates, using three-four negative control samples in each experiment (representative examples are shown on Fig. 2). For regions tested using both of the above methods, results were in concordance with each other.

### Mutation nomenclature

Naming of the variants complies with the recommendations of the Human Genome Variation Society [37, 38]: sequence changes are named in relation to the longest cDNA reference sequences (NM_000038.5 for APC and NM_001128425.1 for MUTYH), while predicted changes at the protein level are given according to the corresponding protein reference sequences (APC: NP_000029.2 and MUTYH: NP_001121897.1).

### Statistics

Differences between groups were calculated by comparison of means using the Student’s *t* test, with p values less than 0.05 considered significant.

## Results

### Patient characteristics

A total of 87 unrelated probands (52 males and 35 females) with familial adenomatous polyposis were included in this study. The median age of diagnoses was 27 years (ranging from 6 to 53 years). The majority of the patients (66/87, 76 %) showed symptoms of profuse, classical polyposis with several hundreds or thousands of polyps in the large bowel, the rest could be classified as attenuated FAP (AFAP) cases with less than 100 adenomatous polyps present. Clinical data on extracolonic manifestations were only rarely available, but five cases were reported to have a known phenotypic variant of FAP (four probands with Gardner syndrome and one with Turcot syndrome). Available clinical and mutation data are summarized in Tables 1, 2 and 3.

**Table 1:** Pathogenic APC variants: substitutions and small indels

Family	Age^a^	Polyp^b^	Pheno-type^c^	Coding exon	Mutation		References
					Nucleotide	Predicted protein	
HFC005	25	++	AFAP	3	c.416_419del4	p.Lys139Argfs*30	[39]
HFC068	43	++	AFAP	3	c.416_419del4	p.Lys139Argfs*30	[39]
HFC201	42	+++	cFAP,G	4	c.423-2A>G	Skipping of exon 4	[40]
HFC127	34	++	AFAP	4	c.423G>T	Skipping of exon 4	[41]
*HFC043*	*44*	+++	*cFAP*	*4*	*c.513_516del4*	p.Pro173*	*Novel*
HFC024	40	+++	cFAP	5	c.556delA	p.Arg186Glufs*19	[42]
HFC122	23	+++	cFAP	6	c.646C>T	p.Arg216*	[43]
HFC079	33	+	AFAP	6	c.694C>T	p.Arg232*	[44]
*HFC137*	*24*	++	*AFAP*	*7*	*c.793G*>*T*	p.Gly265*	*Novel*
HFC044	24	+++	cFAP	13	c.1690C>T	p.Arg564*	[45]
HFC135	29	+++	cFAP	13	c.1690C>T	p.Arg564*	[45]
HFC224	35	+++	cFAP	13	c.1738A>T	p.Lys580*	[46]
*HFC223*	*16*	+++	*cFAP*	*14*	*c.1748dupC*	p.Thr584Asnfs*18	*Novel*
HFC144	22	+++	cFAP	14	c.1886delT	p.Leu629*	[47]
HFC062	43	++	AFAP	14	c.1957A>G	Skipping of exon 14	[41]
HFC082	36	+++	cFAP	14	c.1957A>G	Skipping of exon 14	[41]
HFC063	44	+++	cFAP	14	c.1958+3A>G	Skipping of exon 14	[41]
HFC015	27	+++	cFAP	15	c.2138C>G	p.Ser713*	[4]
*HFC249*	*18*	+++	*cFAP*	*15*	*c.2186dupT*	p.Met730Hisfs*4	*Novel*
HFC129	27	+++	cFAP	15	c.2387_2388delAT	p.Tyr796Trpfs*2	[48]
HFC194	26	+++	cFAP	15	c.2413C>T	p.Arg805*	[49]
*HFC095*	*34*	+++	*cFAP*	*15*	*c.2545delG*	p.Asp849Ilefs*12	*Novel*
HFC070	25	+++	cFAP	15	c.2626C>T	p.Arg876*	[50]
HFC217	21	+++	cFAP	15	c.2626C>T	p.Arg876*	[50]
HFC170	29	+++	cFAP	15	c.2805C>G	p.Tyr935*	[51]
*HFC026*	*27*	+++	*cFAP*	*15*	*c.2973_2976del4*	p.Lys993Phefs*11	*Novel*
*HFC240*	*30*	+++	*cFAP*	*15*	*c.2984delG*	p.Cys995Serfs*10	*Novel*
HFC020	27	+++	cFAP,T	15	c.3183_3187del5	p.Gln1062*	[44]
HFC036	36	+++	cFAP	15	c.3183_3187del5	p.Gln1062*	[44]
HFC221	15	+++	cFAP	15	c.3183_3187del5	p.Gln1062*	[44]
HFC222	23	+++	cFAP	15	c.3183_3187del5	p.Gln1062*	[44]
HNF001	38	+++	cFAP	15	c.3183_3187del5	p.Gln1062*	[44]
HFC008	16	+++	cFAP	15	c.3186_3187delAA	p.Ser1063*	[47]
*HFC101*	*19*	*NA*	*cFAP*	*15*	*c.3203_3206del4*	p.Ser1068*	*Novel*
*HFC061*	*17*	++	*AFAP*	*15*	*c.3257_3258delAC*	p.His1086Profs*32	*Novel*
*HFC065*	*39*	+++	*cFAP*	*15*	*c.3257_3258delAC*	p.His1086Profs*32	*Novel*
HFC099	18	+++	cFAP	15	c.3427delT	p.Tyr1143Ilefs*22	[52]
HFC067	10	+++	cFAP	15	c.3471_3474del4	p.Glu1157Aspfs*7	[53]
HFC033	28	+++	cFAP	15	c.3473_3474delGA	p.Arg1158Thrfs*5	[54]
HFC212	30	+++	cFAP	15	c.3682C>T	p.Gln1228*	[47]
HFC080	15	+++	cFAP	15	c.3880C>T	p.Gln1294*	[55]
HFC042	19	+++	cFAP	15	c.3925_3928del4	p.Glu1309Argfs*11	[56]
HFC030	32	+++	cFAP	15	c.3927_3931del5	p.Glu1309Aspfs*4	[44]
HFC038	13	+++	cFAP	15	c.3927_3931del5	p.Glu1309Aspfs*4	[44]
HFC040	17	+++	cFAP	15	c.3927_3931del5	p.Glu1309Aspfs*4	[44]
HFC152	17	+++	cFAP	15	c.3927_3931del5	p.Glu1309Aspfs*4	[44]
HFC155	24	+++	cFAP	15	c.3927_3931del5	p.Glu1309Aspfs*4	[44]
*HFC233*	*20*	+++	*cFAP*	*15*	*c.3939delT*	p.Arg1314Glyfs*7	*Novel*
HFC089	6	+++	cFAP	15	c.4057G>T	p.Glu1353*	[57]
HFC187	24	+++	cFAP	15	c.4067C>A	p.Ser1356*	UMD.be^d^
HFC041	15	++	AFAP	15	c.4099C>T	p.Gln1367*	[58]
*HFC088*	*11*	+++	*cFAP*	*15*	*c.4268_4271del4*	p.Leu1423Glnfs*49	*Novel*
HD001	19	+++	cFAP,G	15	c.4348C>T	p.Arg1450*	[59]
HFC213	42	+++	cFAP	15	c.4348C>T	p.Arg1450*	[59]
*HFC200*	*27*	+++	*cFAP,G*	*15*	*c.4426delG*	p.Val1476Phefs*31	*Novel*
HFC018	20	+++	cFAP,G	15	c.4549C>T	p.Gln1517*	[60]

Novel mutations are shown in *italics*

* Predicted Protein column are part of the official HGVS nomenclature (denoting STOP codon)

^a^Age of onset

^b^Number of polyps found at diagnosis (+: <30; ++: 30–100; +++: >100; NA: not available)

^c^cFAP: classical FAP; AFAP: attenuated FAP; G: Gardner syndrome; T: Turcot syndrome

^d^Listed in the APC mutation database at [http://www.umd.be/APC/] without reference

**Table 2:** Pathogenic APC variants: large deletions

Family	Age^a^	Polyp^b^	Exon	Mutation name	Repetitive elements^c^
HFC132	42	++	4	c.423-1108_532-2193del4086	AluJb/AluSc8
HFC220	18	+++	13–14	c.1627-185_1958+651del7146insGATCCT	AluJr4/AluSz
HFC126	24	+++	14	c.1744-2465_1958+976del3656insGTAA	Non-Alu/AluSx1
HFC145	45	+++	0–13	c.1-?_1743+?del	Not determined
HFC184	22	++	0–13	c.1-?_1743+?del	Not determined
HFC203	32	++	0–13	c.1-?_1743+?del	Not determined
HFC110	13	+++	14–15	c.1744-?_8532+?	Not determined
HFC106	26	+++	0–15	c.1-?_8532+?del	Not determined
HFC208	33	+++	0–15	c.1-?_8532+?del	Not determined

^a^Age of diagnosis

^b^Number of polyps found at diagnosis (++: 30–100; +++: >100)

^c^Repetitive elements located at the 5′/3′ breakpoints according to the RepeatMasker software [http://www.repeatmasker.org/]

**Table 3:** Biallelic MUTYH mutations

Family	Age^a^	Polyp^b^	Exon	Mutation name	Consequence (predicted)	References
HFC238	50	+++	5	c.504+19_504+31del13	p.Lys155_Glu168del	[61]
			9	c.734G>A	p.Arg245His	[62]
HFC006	51	++	6	c.453_458dup	p.Thr152_Met153insIleTrp	[17]
			9	c.734G>A	p.Arg245His	[62]
HFC185	35	+++	6	c.453_458dup	p.Thr152_Met153insIleTrp	[17]
			10	c.933+3A>C	p.Gly264Trpfs*7	[63]
HFC239	30	++	7	c.536A>G	p.Tyr179Cys	[15]
			9	c.734G>A	p.Arg245His	[62]
HFC049	46	+++	9	c.734G>A	p.Arg245His	[62]
			9	c.734G>A	p.Arg245His	

^a^Age of onset

^b^Number of polyps found at diagnosis (++: 30–100; +++: >100)

### Mutations of the APC gene

**Fig. 3:**
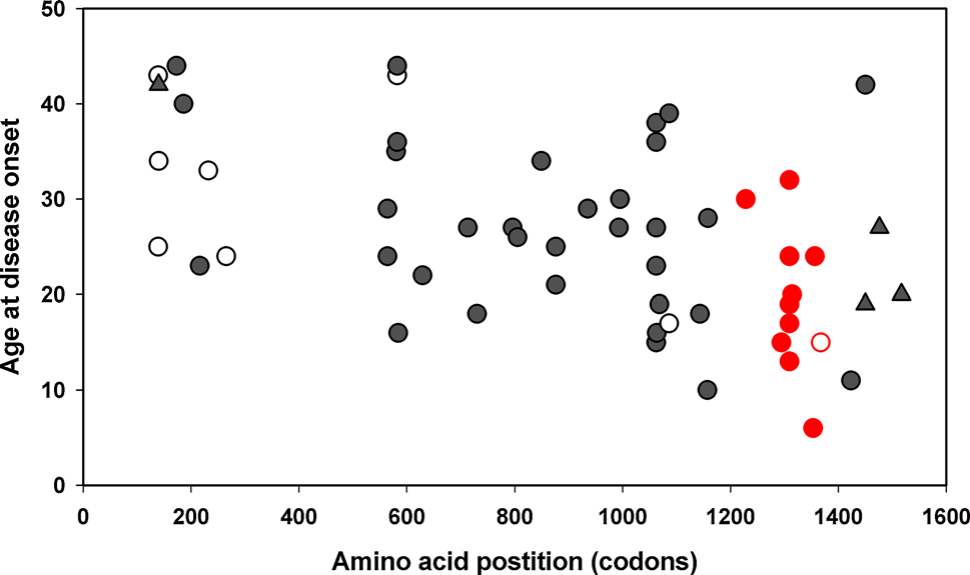
Genotype–phenotype correlations for substitution and small indels of the APC gene. The age of disease onset is shown in relation to the position of the mutated codon. For splice site mutations resulting in exon skipping, the last codon of the previous exon was used. To demonstrate genotype–phenotype correlations, mutations found in AFAP cases are depicted as *empty symbols* (clustering near the 5′ part of the gene), while the mutations of patients diagnosed with Gardner syndrome are shown as *triangles* (mostly after codon 1400). *Red symbols* are used to highlight the mutations within the codon 1200–1400 region, their carriers showing a significantly reduced age at disease onset as compared to the carriers of mutations located elsewhere in the gene. (Color figure online)

Mutation analysis using direct sequencing and MLPA revealed the presence of a deleterious sequence variant in the APC gene in 75 % of the probands (65/87), with 52 % (11/21) among the AFAP patients and 82 % (54/66) among classical FAP probands. The mutation spectrum consists of nine genomic deletions (14 %), and 56 point mutations. Three of the large deletions could be localized with both breakpoints within the APC gene and involving Alu repeat elements (Fig. 1), while the other six extended over the gene boundaries, half of them affecting neighbouring genes, two of them containing the entire APC sequence (Figs. 2, 3). Of the 56 point mutations, 32 were small indels, 19 were nonsense substitutions and 5 were variants predicted to lead to altered splicing. The point mutations represent 42 different pathogenic changes, 12 (29 %) of them novel, one seen in two reportedly unrelated families. The most frequently occurring mutations (c.3183_3187del5; p.Gln1062* and c.3927_3931del5; p.Glu1309Aspfs*4) were seen in five probands each. A summary of the mutations found in our patients is given in Tables 1 and 2.

### Biallelic MUTYH mutations

Twenty-two patients without evidence of pathogenic APC variants were analyzed for mutations in the MUTYH gene. Direct sequencing revealed five cases (23 %) with biallelic MUTYH mutations, three carriers with the classical FAP phenotype and two from the AFAP group. None of the mutations were novel (Table 3).

### Genotype–phenotype correlations

Patients with constitutional APC mutations showed a median age of 26 years (range 6–45 years) at disease onset, which significantly differed (*p* = 0.018) from that of the mutation negative cases (median 37 years, ranging from 7 to 53 years), and those with biallelic MUTYH mutation (median 46 years ranging from 35 to 51 years *p* = 0,029). Biallelic MUTYH mutation carriers showed no difference compared to mutation negative cases (*p* = 0.084).

Extracolonic manisfestations were rarely reported, but three of the four Gardner syndrome cases described here (diagnosed with multiple adenomas together with desmoid tumors) were found to carry an inactivating APC mutation after codon 1400. The majority of AFAP cases were found to carry a pathogenic APC mutation located in the 5′ part of the gene (Table 1; Fig. 3). Regarding the association of the mutation position with the patients’ age at onset we demonstrated a significantly reduced age for those carrying a mutation in the 100 amino acid vicinity of codon 1300 as compared to those with mutations in other parts of the APC gene (*p* < 0.003) (Fig. 3).

## Discussion

There is only limited information of the spectrum of APC and MUTYH mutations in the Central-Eastern-European region [20–25]. In this study the coding region of the APC gene has been screened for mutations in a panel of 87 unrelated probands diagnosed with familial adenomatous polyposis. The methods applied for mutation analysis included direct sequencing and also screening for copy number alterations using MLPA, and this combined approach allowed us to identify a pathogenic alteration in 75 % of the patients analysed, with 82 % of the classical FAP cases.

The scattered mutation pattern, the marked predominance of small deletions (more than half of the point mutations falling into that category) and also the relative frequency of large genomic alteration (14 %) is in agreement with most previous findings in European populations [64–67]. The frequency of the two most commonly identified mutations at codon hot spots 1062 and 1309 in our sample set was 12 % each, which falls into the same range as reported by several other groups [66, 68, 69].

From the total of 42 unique point mutations, 12 were novel (29 %), which is in concordance with the wide range of this type of alterations found in different populations (from 16 % in Koreans to >40 % in Northern Europe) [52, 70, 71] and also with the frequencies appearing in the Human Gene Mutation Database. This relatively high frequency of novel alterations underscores the need of screening different populations in order to reveal their possibly distinct mutational spectra.

Detection of large genomic alterations are recently included more often in the routine mutation screening protocols then before, but the detailed characterization of these changes requires more time and dedicated techniques, which usually are outside the capacity of most laboratories. Thus, large genomic deletions are rarely studied in detail [64, 67, 72], although at least some of them may extend into neighbouring regions which potentially have a modifier effect on the disease phenotype as exemplified by some recent studies including our group [29, 32, 73–75]. In our series of cases, nine large genomic deletions were identified, varying widely in size, ranging from single exon deletions to an extremely large deletion containing many genes.

Three of the large genomic alterations had both breakpoints located within the gene (deletion of exon 4, exon 14, and exons 13–14). For these cases we were able to determine the exact breakpoints, revealing a role of repetitive elements: different Alu sequences were involved in all cases, and in two instances a 4–6 bp non-template insertion at the breakpoint junction was also observed, indicating the classical non-homologous end joining (NHEJ) as the most likely mechanism responsible for these deletions [76–80].

**Fig. 4:**
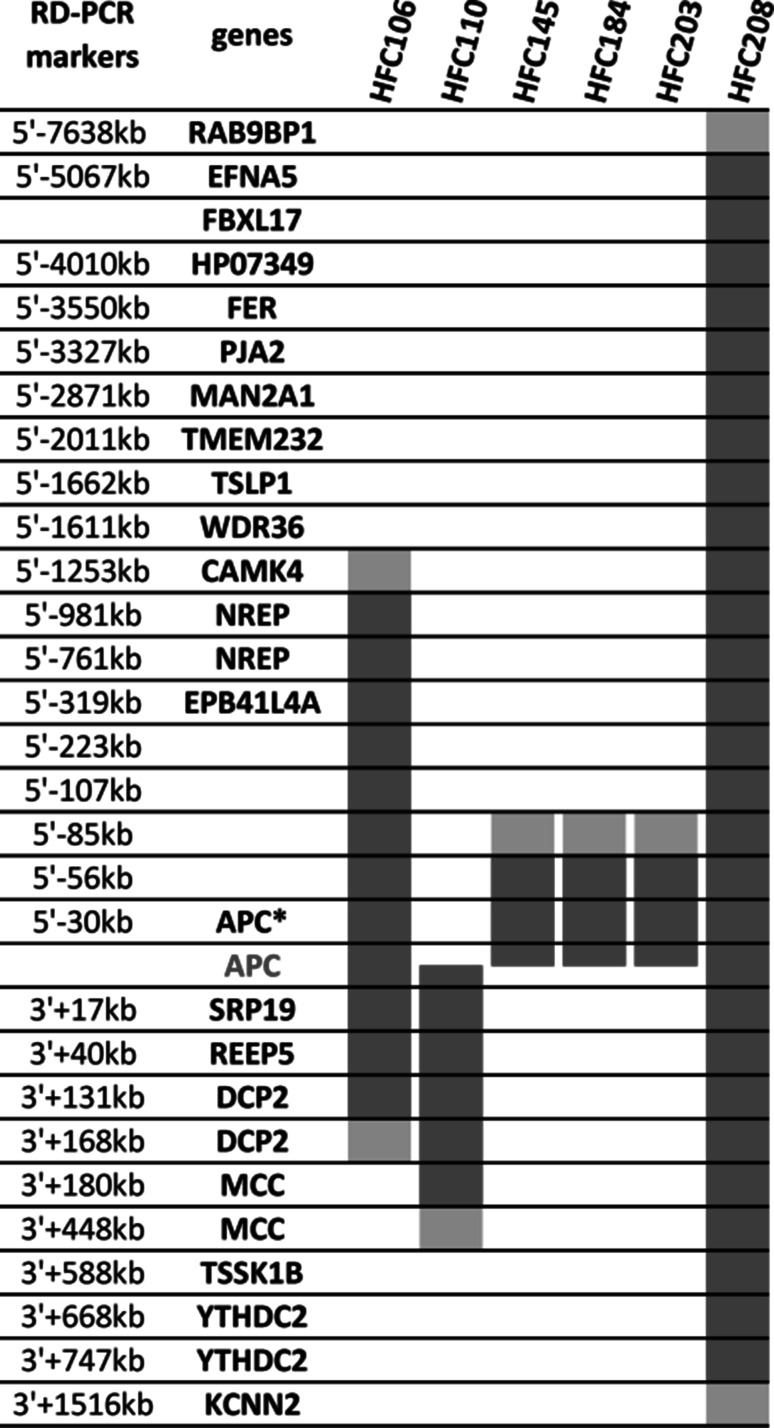
Germline large deletions extending over the boundaries of the APC gene. The localization of the known RefSeq genes of the chromosome 5 region 104,000,000–114,000,000 (coordinates are given according to the GRCh37/gh19 chromosome assembly) are shown schematically. The minimal and maximal sizes of the large genomic deletions are indicated for our six samples as *red* and *pink bars*, respectively. The loci where copy number analyses were done (RD-PCR markers) are shown on the *left*, their names reflecting their localization with respect to the coding portion of the APC gene. APC*: the 5′–30 kb RD-PCR marker is located in the first non-coding exon of APC. *Markers without gene names* are in intergenic regions. (Color figure online)

The remaining six deletions extended over the gene boundaries, half of them reaching other upstream and/or downstream genes. The exact breakpoints were not specified, but we applied two independent semiquantitative techniques to determine the copy numbers (gene dosage) in several regions outside the APC gene, thus mapping the approximate sizes of these mutations. One of them (sample HFC208) was found to be more than 4 MB long and also affected the coding regions of several genes up- and downstream of APC (Fig. 4). Although some of these genes were indicated in colorectal carcinogenesis [2, 81, 82], their heterozygous deletion did not seem to cause any modification in the polyposis and/or CRC phenotypes of the probands carrying them. However, given the small number of patients carrying such large genomic deletions in our study, a considerably larger dataset would be required to reliably assess the potential role of these neighbouring genes.

From the 22 FAP/AFAP patients found negative for germline APC mutations, biallelic MUTYH mutations accounted for five (23 %) cases, increasing the overall mutation detection rate to 80 %. Of the two mutations most frequently reported in the literature to date, p.Tyr179Cys and p.Gly396Asp, (responsible for ~80 % of pathogenic variants found in European populations [83]), only the former was found in one case, emphasizing the need to determine the possibly characteristic population-specific mutation patterns by a comprehensive screening of the whole coding sequence for the gene for all populations studied.

Understanding genotype–phenotype correlations is useful for the clinical management of (A)FAP families [8–10], but the relationships between the locations of the *APC* mutations and certain extracolonic manifestations are still not fully delineated, and as more patients are diagnosed with (A)FAP, a broader range of extracolonic manifestations has come to be recognized in this group. For our sample set the extracolonic manifestations were rarely reported, so the only statistically significant correlation we could demonstrate was the association between the age of disease onset and the location of the APC mutation in the codon 1200–1400 region, which is in agreement with several previous reports [46, 60, 84–87].

The inherent variability of the (A)FAP phenotype and also the overlap between APC- and MUTYH-linked phenotypes (that is, MUTYH mutations can be associated with classical FAP features) necessitate a more comprehensive approach for FAP screening to increase mutation detection yield. A combined analysis of the two genes with techniques allowing for the detection of both point mutations and large genomic alterations is needed for the exhaustive mutation testing of both classical FAP and attenuated FAP cases [13, 88, 89].

Finally, our patient series includes 17 families with no germline APC or MUTYH mutation detected, although ten of them belong to the classical FAP group with profuse polyposis. In these cases the age of disease onset was significantly older than that of the APC mutation carriers (34.5 vs. 26.7 years, *p* = 0.018), and almost 10 years younger than those with biallelic MUTYH mutations (44 years). Since APC/MUTYH-negative cases are noticeably enriched in AFAP patients, while the mutation positive group in classical FAP cases, we also compared age of onset data separately for the FAP and AFAP groups, and found no significant difference between mutation negative and positive cases (*p* = 0.18 for the AFAP and *p* = 0.3 for the FAP group). Mutation negative cases raise the possibility of yet uncovered genetic heterogeneity of FAP, the possible role of other predisposing genes [90, 91], but also the incompleteness of the routinely used mutation screening techniques: ignored, but potentially regulatory regions in introns or even outside the gene boundaries may also contribute to inactivation of a predisposing gene [73, 92–96].
